# Nanophotonic sensing and label-free imaging of extracellular vesicles

**DOI:** 10.1038/s41377-025-01866-2

**Published:** 2025-04-28

**Authors:** Isabel Barth, Hakho Lee

**Affiliations:** 1 Center for Systems Biology, Massachusetts General Hospital, Harvard Medical School, Boston, MA 02114 USA; 2 Department of Radiology, Massachusetts General Hospital, Harvard Medical School, Boston, MA 02115 USA

**Keywords:** Applied optics, Optical sensors

## Abstract

This review examines imaging-based nanophotonic biosensing and interferometric label-free imaging, with a particular focus on vesicle detection. It specifically compares dielectric and plasmonic metasurfaces for label-free protein and extracellular vesicle detection, highlighting their respective advantages and limitations. Key topics include: (i) refractometric sensing principles using resonant dielectric and plasmonic surfaces; (ii) state-of-the-art developments in both plasmonic and dielectric nanostructured resonant surfaces; (iii) a detailed comparison of resonance characteristics, including amplitude, quality factor, and evanescent field enhancement; and (iv) the relationship between sensitivity, near-field enhancement, and analyte overlap in different sensing platforms. The review provides insights into the fundamental differences between plasmonic and dielectric platforms, discussing their fabrication, integration potential, and suitability for various analyte sizes. It aims to offer a unified, application-oriented perspective on the potential of these resonant surfaces for biosensing and imaging, aiming at addressing topics of interest for both photonics experts and potential users of these technologies.

## Abstract (web summary)

This review examines imaging-based nanophotonic biosensing and interferometric label-free imaging, with a particular focus on vesicle detection. It specifically compares dielectric and plasmonic metasurfaces for label-free protein and extracellular vesicle detection, highlighting their respective advantages and limitations.

## Graphical abstract

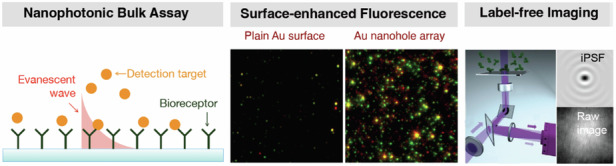

## Introduction

Protein assays are essential tools in basic and clinical research^1^. Detecting specific protein targets is key to identifying disease biomarkers, elucidating disease mechanisms, and evaluating the efficacy of new therapeutics, which, in turn, advances clinical diagnosis, prognosis, and interventions^2,3^. For instance, measuring circulating proteins in the blood (e.g., prostate-specific antigen, cancer antigen 125) has implications for detecting tumor recurrence and monitoring treatment response^4,5^. Recent progress in extracellular-vesicle (EV) research further underscores the importance of protein detection^6^. EVs are nanoscale biological particles secreted by cells into the circulation, carrying protein cargos reflective of a cell of origin and physiological state^7^. Detecting EV proteins has demonstrated potential for generating valuable clinical information, including tumor origin, progression, and treatment outcomes^8–10^. These markers are readily accessible in bodily fluids, providing a minimally invasive and repeatable means to complement tissue biopsies and medical imaging^11^.

Conventional protein assays frequently employ fluorescent or enzymatic labeling to generate analytical signals. A common example is the enzyme-linked immunosorbent assay (ELISA), which typically utilizes a pair of antibodies: one immobilized on a substrate to capture target proteins and the other labeled with signaling molecules for detection^12,13^. This dependence on matched antibody pairs in ELISA restricts its applicability when suitable pairs are unavailable^14^. Moreover, the labeling process itself carries the risk of altering the structure or function of the target biomolecules and increases assay complexity and cost^15^. Label-free detection methods may offer a solution to these technical challenges^16,17^. Techniques such as surface plasmon resonance (SPR), photonic crystal resonance, interferometry, and nanoparticle tracking analysis enable the direct study of biomolecules in their native state, avoiding potential labeling artifacts^18,19^. These methods could be valuable for detecting and characterizing EVs, which are heterogeneous and often present at low concentrations in biological samples. Importantly, label-free approaches facilitate real-time analysis, providing biological insights that are not readily accessible through traditional methods while also offering faster, higher-throughput capabilities^20^.

**Fig. 1:**
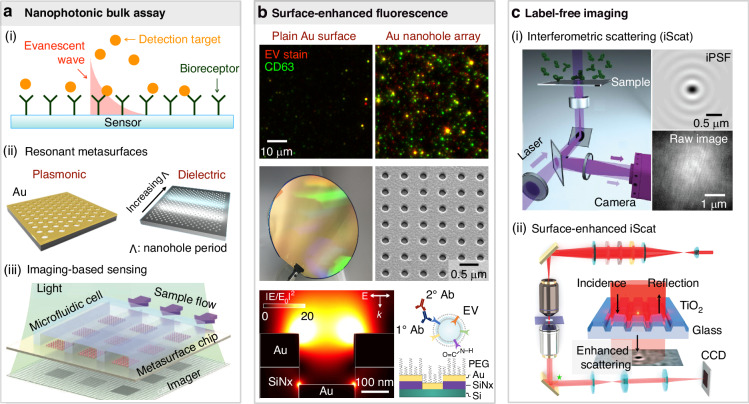
Outline of the review with separate strands for sensing (bulk assays) and imaging (single particles). **a** Nanophotonic sensing of bulk analytes. (i) General schematic of biosensing based on evanescent waves. (ii) Examples of resonant metasurfaces, including plasmonic nanohole arrays (left) and dielectric nanohole arrays (right, shown in a ‘chirped’ configuration). (iii) The readout can take many forms, such as spectral or intensity-based approaches. We limit this review to imaging-based sensing. Images are adapted from^25,44,106,107^. **b** Single particle imaging using fluorescence microscopy. Resonant surfaces can be used to enhance fluorescent signals from single particles. Subfigures rearranged and taken from^92^. **c** Label-free imaging. (i) Interferometric scattering microscopy (iScat) can detect single particles without labels. (ii) Resonant metasurfaces are used for surface-enhanced iScat. Images are from^108,109^. Ab, antibody; PEG, polyethylene glycol; iPSF, interferometric point spread function; CCD, charge-coupled device

This review explores the emerging role of imaging-based nanophotonic sensing and interferometric label-free imaging in EV detection (see outline in Fig. 1), focusing on the comparative strengths of dielectric and plasmonic metasurfaces. By bridging insights from photonics and biology, we aim to highlight the potential of these technologies for advancing biomedical research and diagnostic applications.

## Imaging-based refractometric sensing with nanophotonic surfaces

This section discusses resonances in dielectric and plasmonic surfaces for the label-free, refractometric sensing of proteins and vesicles. Refractometric sensing implies that a target analyte is detected by monitoring a local change in the effective refractive index of the resonant mode on the sensor surface. The evanescent-field enhancement associated with nanophotonic and plasmonic resonances can enable the label-free detection of proteins^21–24^ and vesicles^25–27^ at clinically relevant concentrations.

Various refractometric read-out schemes are suitable for quantifying the concentration of proteins and vesicles. We here restrict our discussion to imaging-based sensing approaches (Fig. 1a). The term *imaging-based* nanophotonic sensing denotes sensing approaches that are different from platforms such as waveguide- and fiber-based sensing technology or any approach employing spectrometers but does not refer to systems enabling imaging of single proteins or vesicles (Fig. 1b), which is covered in the next section on single particle imaging. The reasoning for this distinction is to highlight the level of integration with microscopy. For example, fiber-based sensors can be employed for remote sensing, and waveguide-based platforms allow the multiplexed detection of proteins. However, these systems are not designed to spatially resolve refractive index distributions or enhance traditional microscopy. The photonic terms for this distinction are *in-plane coupling* (waveguides) versus *out-of-plane coupling* (dielectric and plasmonic resonant surfaces). It is this out-of-plane coupling aspect that allows the straightforward, collinear integration of nanophotonic surface platforms into conventional microscopes, and it is the resulting imaging aspect that enables a parallel implementation of references, controls, and detection of biomarkers with a camera-based single-shot read-out.

There are two main categories of resonant surfaces for refractometric sensing for collinear optical integration: (i) dielectric nanostructured surfaces based on materials such as silicon nitride (Si_3_N_4_), silicon (Si), or titania (TiO_2_) supporting photonic crystal resonances and (ii) plasmonic nanostructured surfaces based on gold or other metals supporting extraordinary optical transmission (EOT). In the following, we overview the recent innovations in both dielectric and plasmonic arrays developed for biosensing. We also compare the differences and advantages of these two approaches depending on the application.

### Dielectric and plasmonic resonant metasurfaces—state-of-the-art discussion

Plasmonic nanohole arrays have reached a high degree of demonstrated applicability in sensing and imaging inside and beyond laboratories in the last decade, where the first publications on integrated gold nanohole arrays for sensing date back to nearly two decades ago^28,29^. Since there exist comprehensive review articles on this topic^27^, we will limit the discussion to the most recent publications related to state-of-the-art, label-free detection of proteins and vesicles.

We focus on the performance achieved with dielectric arrays, which first demonstrated label-free sensing capabilities for biochemical assays in 2002^30^. These dielectric platforms are essentially photonic crystal slabs^31^. The resonances that they support are called, e.g., guided-mode resonances^32^ or resonant leaky modes^33^, and have been described under various technical frameworks, such as high-contrast subwavelength gratings, non-local metasurfaces, all-dielectric metasurfaces and, increasingly, (quasi) bound states in the continuum (BIC).

Plasmonic sensors may generally be perceived to offer higher performance than dielectric platforms^34^: dielectric nanohole arrays lack the strong field enhancement and confinement provided by plasmonic effects. Consequently, dielectric arrays may have lower sensitivity and higher detection limits compared to their plasmonic counterparts. The main advantage of dielectric arrays may lie in fabrication simplicity and cost in some cases, as well as the smaller effect of photothermal heating compared to plasmonics^35^. We critically examine these assumptions, comparing plasmonic and dielectric extended arrays (e.g., arrays of nanoholes, pillars, ellipses, and other shapes) that allow an imaging-based read-out through out-of-plane coupling.

**Fig. 2:**
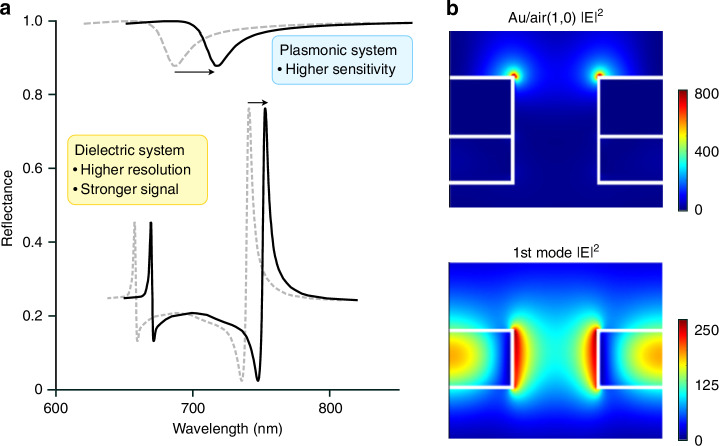
Comparison between plasmonic and dielectric nanohole arrays. **a** Schematic of typical spectra. The plasmonic resonance spectrum (dashed line) is based on experimental results in ref. ^65^, and the dielectric resonance spectrum (dashed line) on experimental results in ref. ^44^. This illustration highlights the practical differences between plasmonic and dielectric metasurfaces for sensing, presenting their respective advantages in terms of resonance Q-factor, amplitude, and sensitivity. The arrows indicate the peak shift towards higher wavelengths with increasing refractive index. This is a conceptual representation illustrating the enhanced refractometric sensitivity of plasmonic systems and is not based on empirical data. **b** The electric field (E) simulations show plasmonic ‘hotspot’ formation with strong near-field enhancement (top) and more extended confinement of resonances in a dielectric array (bottom). The image was adapted from^47^

A side-by-side comparison of the features defining resonances in dielectric and plasmonic nanostructured surfaces (Fig. 2a) shows clear differences in the resonance amplitude and the quality factor (Q) as well as in the evanescent field enhancement. Plasmonic resonances have a lower amplitude (peak signal strength) and Q (wavelength/peak width) mainly because of the absorption losses associated with the involved metal layers in the visible wavelength range. In contrast, dielectric materials can be transparent, and nearly 100% reflectance is achievable with resonances supported by dielectric surfaces. Actual reflectance values are typically lower, not from absorption in the material but from scattering losses due to surface roughness and fabrication imperfections, which is especially relevant for high-Q resonances^36^. On the other hand, typical plasmonic resonance peaks show considerably lower amplitudes^37,38^ (~10%).

For biochemical sensing, a higher amplitude (strong signals) and Q-factor (sharp peaks, high resolution) both contribute to a superior sensing performance^36^. A high Q-factor is beneficial since it can support either high spectral accuracy in resonance peak tracking^39^ or high sensitivity in intensity/phase readout^40–42^. These are the advantages of dielectric platforms, which become especially relevant when the chosen read-out method leverages them, such as in amplitude- and phase-sensitive^21,42,43^ approaches or those that use spatially tuned nanostructures to translate a narrow resonance peak into high sensitivity^22,44–46^.

On the other hand, the advantages of plasmonic metasurfaces become apparent when analyzing the sensitivity and the evanescent field enhancement (Fig. 2b). An experimental comparison of dielectric and plasmonic nanohole arrays suggests an at least two times higher sensitivity of the plasmonic array than that of the dielectric platform^47^. The relationship between sensitivity and near-field enhancement is sometimes attributed to the strength of the near-field enhancement: one would interpret a strong near-field enhancement resulting in a high sensitivity^48^. However, the reasons for differences in sensitivity are not based on the absolute enhancement values but on the degree of *overlap* of the evanescent field with the analyte.

Photonic crystal resonances in periodic dielectric nanostructures have been used for a variety of sensing applications^49^. At certain resonance conditions, the coupling of the guided mode to external plane waves, leading to the leaky nature of these resonances, can be suppressed by a symmetry-mismatch between the guided mode and the continuum or destructive interference^50^; this can lead to theoretically infinite lifetimes of the resonances^51^. This phenomenon has been studied since the early 21^st^ century^52–55^, and was recently included in the broader classification of photonic BIC^50^. The potential of photonic BIC for biosensing has already been predicted in 2008^56^ and the study of experimentally feasible, asymmetric metasurfaces supporting quasi-BIC^51^ has led to a transfer of these potentially high Q-factor resonances from theoretical studies to practical sensing applications in recent years^48,57^. Quasi-BIC and guided-mode/photonic crystal resonances in dielectric metasurfaces/photonic crystal slabs are governed by the same physics based on the coupling of guided modes to the radiating continuum, and both show a characteristic Fano-shape of their resonance peaks^51^. We discuss the quasi-BIC phenomena here because the potential to reach infinite Q-factors provides an interesting starting point for the critical discussion of experimentally verified performance and the comparison to theoretical design.

**Fig. 3:**
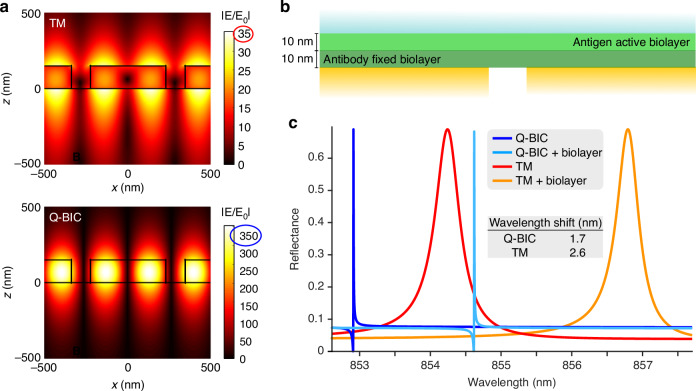
Numerical simulation of resonances in periodic dielectric arrays. **a** The Electric field (E), normalized to an incident field (E_0_), was calculated for a silicon nitride photonic crystal slab. The top panel shows photonic crystal resonance (TM), and the bottom panel shows a symmetry-protected bound state in the continuum (BIC). Breaking the symmetry via a nonzero incidence angle (here 0.1°) results in a quasi-BIC (Q-BIC) mode with high Q and a ~ 10-fold higher enhancement in the electric field magnitude (compare the values inside red and blue ellipses). **b** Schematic representation of a biosensing configuration, illustrating the binding of target proteins (antigen active biolayer) to a functionalized surface (antibody fixed biolayer). **c** Calculated resonance spectra of the silicon nitride photonic crystal slab. Both TM and Q-BIC modes exhibit a redshift upon biolayer addition. The TM mode exhibits a more significant shift (i.e., higher sensitivity) compared to the Q-BIC mode, which is attributed to a greater field overlap with the analyte volume

For photonic crystal resonances, including quasi-BIC, supported by dielectric arrays, a higher Q-factor leads to a stronger field enhancement. However, when the field is confined to the high-index material rather than extending into the surrounding medium (Fig. 3), there is a lower degree of overlap with the analyte; this diminishes the device’s sensitivity to refractive index changes on its surface and consequently, the figure of merit. Refractive index sensitivity, thus, should be attributed to the accessibility of the near-field rather than an absolute field enhancement^25^. A refined characterization^31^ considers the specific analyte characteristics and optimizes the overlap of the field with the target. Since possible target analytes can vary from small molecules to proteins to vesicles or even cells, ranging from <1 nm to tens or hundreds of nm to micron-sized objects, an application-informed “detection zone”^58^ needs to be considered, where the length of the evanescent tail would ideally match the size of the target particles. In simplified terms, plasmonic platforms with strongly confined and localized fields (hotspots or individually addressable silicon nanoantennas^59^) are better suited for the detection of small analytes than non-local photonic crystal resonances. When detecting larger particles such as EVs, the more extended near-field confinement of certain dielectric arrays can provide an advantage.

### Comparison of figure-of-merits

Overall, the figure-of-merit (FOM = Sensitivity × Q) of plasmonic and dielectric sensors is typically considered comparable on average since the lower sensitivity of the dielectric platforms is compensated for by the narrower linewidth of their resonance peaks. This conclusion is based on a generalized example of a photonic crystal resonance in a dielectric nanohole array with a bandwidth of >10 nm. However, considerably lower bandwidths have been reached experimentally in photonic crystal systems, e.g., with transverse magnetic (TM) guided-mode resonances^42,44^ (Q-factor ~1000) or by using quasi-BIC^50,60^. In contrast, the highest achievable Q-factors for plasmonic resonances typically correspond to experimental linewidths of ~ 25 nm (Q-factor ~25)^61^. These observations raise two key questions: (i) what is the highest FOM experimentally achieved by dielectric and plasmonic platforms, and (ii) do higher Q-factors in dielectric platforms lead to experimentally observable advantages in the FOM compared to plasmonics?

**Fig. 4:**
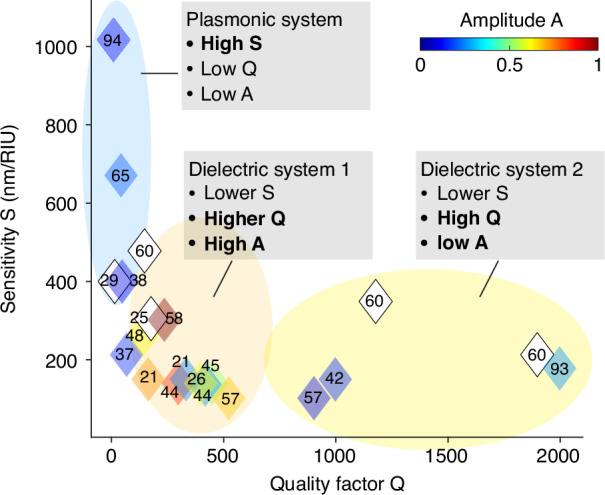
Summary of published state-of-the-art in quality factor (Q), sensitivity (S), and resonance peak amplitude (A). The numbers within the diamond represent the reference numbers from which these values were obtained. Filled (colored) diamonds indicate that A values are directly or indirectly reported in the literature. Unfiled (clear) diamonds indicate that A values were not reported. Plasmonic resonances generally exhibit low Q and A but high S when compared to dielectric array resonances (high Q and A, low S). Some dielectric array resonances demonstrate exceptionally high Q, albeit often at the expense of reduced A. RIU, refractive index unit

Figure 4 summarizes the experimentally determined FOM of existing dielectric and plasmonic systems while also considering the resonance amplitude as an additional aspect^36^. The contrast is apparent between plasmonic sensors (high refractive index sensitivity S) and dielectric platforms (higher Q-factor and a higher resonance amplitude A), which confirms an intuitively predicted behavior based on the typical differences shown in Fig. 2a. Also noticeable is the trend of a lower resonance amplitude of dielectric platforms when their Q-factors reach significantly high values (>1000). The literature search found a limited number of high-Q dielectric systems demonstrated for sensing, suggesting that achieving Q-factors exceeding a certain threshold may not be feasible. This can be attributed to the significant reduction in the resonance amplitude, which leads to low SNR and high read-out noise, particularly when the resolution of the read-out method, such as a spectrometer, is not optimally adapted to the predicted bandwidth of the resonance peak. The lower SNR and consequent noise may negate the advantages of an improved spectral resolution of high-Q resonance peaks. Although the reasoning behind the design of a dielectric platform is rarely discussed in these experimental works, Fig. 4 implies that the loss in the resonance amplitude that accompanies higher Q-factors^36^ leads to designs with a lower Q-factor than the system would theoretically allow. In other words, the designed radiative Q-factor is apparently adjusted to the anticipated non-radiative Q-factor to achieve feasible resonance amplitudes in experiment, accounting for the constraints imposed by the nanofabrication and the optical system.

Intriguingly, a recent work reported both high Q-factors and high sensitivity in dielectric platforms, thereby reaching the best possible FOM^60^. The system utilized quasi-BIC, with an optimized detection zone that maximized the overlap of high-field regions. This approach enabled pairs of high-sensitivity (S) and high-Q factors; S ~ 350 nm/RIU and Q ~ 1000; or S ~ 480 nm/RIU and Q ~ 150. These results, while still reflecting the trade-off between sensitivity and Q-factor, highlight the possibility of leveraging high-Q modes while enabling high sensitivity simultaneously. The proof-of-principle protein sensing used a spectrometer for signal readout (0.02 nm resolution, resonance peak at 1.2 µm). However, the resonance amplitude values are unavailable in the report, making it difficult to assess the feasibility of imaging-based implementations.

### Nanophotonic devices for EV detection

Nanophotonic biosensors are promising tools for EV detection in diagnostic applications. These biosensors offer high sensitivity, label-free detection capabilities, and the potential for miniaturization. A key advancement is the adaptation of surface plasmon resonance (SPR) sensors for EV detection^62^. Nanohole arrays have been incorporated into SPR sensor modalities to enhance sensitivity and allow for a simple integration in conventional microscopes. Silicon photonics biosensors with devices, such as ring resonators and interferometers, have been developed for the ultrasensitive detection of biomolecules, including EVs. These sensors can be integrated into compact lab-on-chip platforms, enabling point-of-care diagnostics. When integrated with microfluidic systems, nanophotonic biosensors facilitated precise sample handling and improved overall sensor performance^63^. Furthermore, artificial intelligence and machine learning are being combined with nanophotonic biosensors to enhance data analysis and improve diagnostic accuracy^64^.

Many examples of clinically relevant protein sensing have been demonstrated with imaging-based nanophotonic platforms^25,42,44,65^. In refractometric sensing, EVs can be viewed as natural nanoparticle tags, making their detection appealing for label-free nanophotonic platforms. However, EV detection with such systems is relatively limited, although EVs have been identified as potential analytical targets^66^. One potential reason for this disparity could be the specialized facilities and expertise required for EV isolation, whereas clinically relevant proteins can be directly purchased from commercial sources to demonstrate the technology’s efficacy. Another contributing factor could be the biological heterogeneity. EVs exhibit significant heterogeneity in marker expression^67^ and size^68^. Bulk assays may mask such diversity, introducing challenges in data interpretation. The research community increasingly embraces single particle analysis as a superior alternative, which is discussed in the following section.

**Fig. 5:**
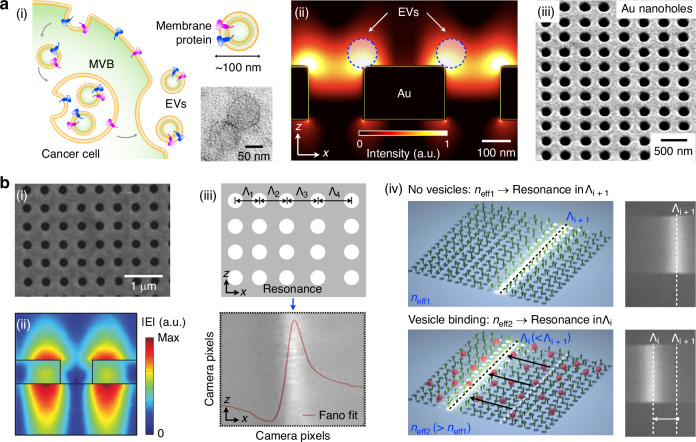
Vesicle detection with metasurface sensors. **a** Label-free detection and molecular profiling of EVs with nanoplasmonic sensor. (i) Cancer cells produce exosomes through the fusion of multivesicular bodies with the plasma membrane. These exosomes carry parental cell proteins. (ii) Simulation showing enhanced electromagnetic fields near a periodic gold nanohole surface. (iii) Scanning electron micrograph of gold nanohole array. Images are adapted from^69^. MVB, multi-vesicular body. **b** Dielectric metasurface for the detection of vesicles^26^ using a ‘chirped’ gradient nanohole array. (i) Scanning electron micrograph of a hydrogenated amorphous silicon nanohole array. (ii) Simulation showing mode confinement of a transverse magnetic (TM) mode with a period of 420 nm, thickness of 120 nm, and radius of 60 nm. (iii) Schematic (top) and image (bottom) of nanohole array with a spatial gradient in its periodicity. The yellow band (top) indicates the resonance, and the red line (bottom) shows a corresponding Fano resonance shape. (iv) Principle of imaging-based vesicle detection with a “chirped” nanohole array with a fixed operating wavelength. Images are adapted from^26,44^

We briefly compare the state-of-the-art nanophotonic sensors employed for the label-free bulk analysis of EVs. Plasmonic sensors (Fig. 5a) have achieved the EV-detection sensitivity down to 670 aM (corresponding to 3 × 10^3^ EV/mL)^69^. A 3D plasmonic photonic crystal biosensor achieved a detection limit of 1 × 10^4^ EV/mL^70^. More recently, dielectric imaging-based platforms based on dielectric nanohole arrays (Fig. 5b) show promise to detect even lower concentrations, down to 10^3^ EV/mL^26^, which is more than 100-fold sensitive than ELISA. As discussed earlier, the detection sensitivity is partly determined by the overlap of the evanescent tail with the target particles. Dielectric metasurfaces thus can be advantageous over plasmonic sensors; dielectric metasurfaces generate a more extended field of guided-mode resonances that closely matches the diameter of EVs (~100 nm), whereas the fields from plasmonic sensors typically match the size of proteins better. Beyond the nanophotonic technology itself, other factors can influence the performance of these sensors. Surface chemistry^71^ and the nature of the sample, such as the presence of a complex matrix versus a buffer, play crucial roles in optimizing and characterizing sensitivity and specificity^72^. In some relevant works^65^, the detection limit is not explicitly stated as concentration, rendering direct comparisons challenging.

### Single nanoparticle imaging—extracellular vesicles

Analyzing biomarkers at the *single* EV level has become important in various preclinical and clinical scenarios. In the context of cancer research, single EV analysis can facilitate the identification of cell or organ-specific EV subpopulations, the detection of scant markers dysregulated in early cancers, and patient monitoring during therapy, particularly for minimal residual diseases^73,74^. Refractometric sensing discussed in the previous section is inherently a bulk EV assay, lacking the power to resolve individual vesicles. Flow cytometry has been adapted to nano-flow cytometry (nFCM), enabling single EV detection with high throughput. However, the read-out variability for low-signal EV samples, e.g., due to inherently high machine noise levels in flow cytometry^75^, the need for further standardization, and the high equipment cost currently hinder a wider instrument adoption.

Microscopy is the predominant analytical technique for single EV visualization and characterization, owing to its superior spatial resolution. Single-EV microscopy can be classified into two categories^76^: (i) label-free methods for assessing EVs’ intrinsic physical properties, including atomic force microscopy, single-particle interferometric reflectance imaging sensors (SP-IRIS), and interferometric nanoparticle tracking analysis^77–79^; and (ii) label-based methods, which employ affinity ligands (e.g., antibodies) conjugated with fluorescent tags^80^ or signal-enhancing nanoparticles^81^ to identify specific molecular targets. SPR imaging^69^ is sometimes classified as a platform for label-free single EV imaging, but this and similar methods^25,26^ are bulk refractive index sensors that do not provide single-particle information. Label-free imaging techniques can detect EVs and provide binary information on the presence of membrane markers by monitoring EV capture on antibody-coated surfaces^78,82^. Examples include SP-IRIS chips with multiplexed capture antibodies^83^, optofluidic biosensors for bulk EV detection^25,26^, and interferometric plasmonic imaging^84^. However, these label-free approaches do not identify multiplexed marker expression at the single-particle level.

### Interferometric scattering microscopy and holography for label-free single EV imaging

**Fig. 6:**
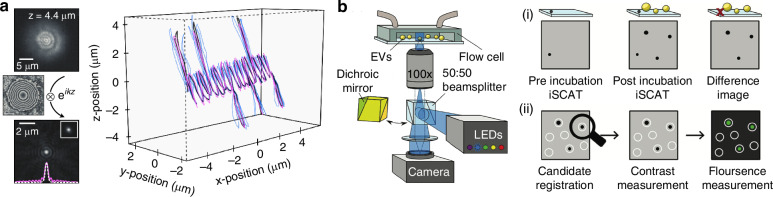
Single EV imaging. **a** Holographic fluorescence imaging of extracellular vesicles. Computational refocusing (left) and 3D tracking of fluorescent beads (right). Images are from^88^. **b** Size photometry of single EVs. Schematic of the microscopy setup with a microfluidic flow cell, EVs on a coverslip, an epifluorescence microscope, sCMOS camera, and a 50:50 beamsplitter for iSCAT imaging. (i) Workflow to suppress background inhomogeneities. A pre-incubation iSCAT image is acquired to capture the background. Subsequently, EVs are immobilized, unbound EVs are washed, and a post-incubation iSCAT image is acquired. (ii) iSCAT images register candidate spots, followed by contrast or fluorescence intensity measurements. Images are from^110^. LED, light emitting diode

Interferometric scattering (iSCAT) microscopy and holography have emerged as potent label-free methods for single EV imaging (Fig. 6). Both modalities offer advantages over fluorescence microscopy, such as longer observation times, faster imaging speeds, and the ability to study EVs in their native state.

iSCAT microscopy utilizes the interference between the scattered light from nanoscale objects and a small diameter reference beam to achieve optimized contrast, thereby enabling a high sensitivity and low limit of detection in particle size^85^. This method has demonstrated the ability to detect and track nanoparticles as small as 5 nm, making it suitable for visualizing individual EVs^86^. Recent advancements include the development of confocal iSCAT^87^ (C-iSCAT), which provides three-dimensional (3D) imaging capabilities and enhanced sensitivity for live-cell studies^88^.

Holographic microscopy captures both the amplitude and phase of light scattered by a sample and reconstructs 3D images. This technique has been successfully applied to track the 3D motion of EVs within live cells, revealing both diffusive and directional transport behaviors. When combined with fluorescence detection, holography microscopy can achieve molecular specificity while preserving the advantages of 3D localization. Recent advancements have incorporated deep learning algorithms for automated cell detection and classification within holographic flow cytometry systems^89^.

Label-free imaging modalities can serve as complementary tools in single-EV analysis, providing physical parameters such as size and refractive index. As such, these modalities augment fluorescence imaging that provides marker-specific molecular information. For instance, interferometric scattering microscopy (iSCAT) has been employed to differentiate EVs from clinically irrelevant particles based on variations in their interferometric point spread function, in conjunction with fluorescence microscopy (Fig. 6b). Integrating these modalities, iSCAT and fluorescence imaging, holds the potential to facilitate novel investigations, for example, exploring potential correlations between marker expression and EV size.

### Single-particle imaging with nanophotonic resonance surface enhancement

**Fig. 7:**
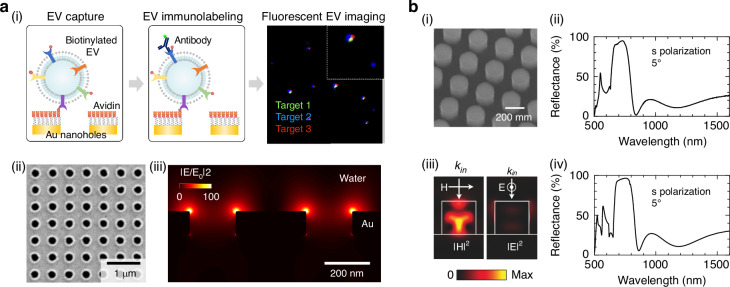
Surface-enhanced fluorescent imaging. **a** Plasmonic nanohole arrays were employed to enhance fluorescent signals in EV imaging. (i) Biotinylated EVs were captured on an avidin-coated gold nanohole array and subsequently labeled with fluorescent antibodies. The assay resulted in enhanced fluorescence single EV imaging. (ii) Scanning electron micrograph of gold nanohole array. (iii) Simulated near-field enhancement of gold nanohole array. Images are adapted from^92^. **b** All-dielectric metasurface fluorescence enhancement for antibody/antigen detection. (i) Scanning electron micrograph of silicon-on-insulator (SOI) nanorod metasurface. (ii) Measured reflectance spectra. (iii) Simulated electromagnetic field intensities at reflectance dip. (iv) Simulated reflectance spectra. Images are adapted from^111^

Nanophotonic surfaces can be used, in conjunction with surface plasmon resonance microscopy (SPRM), to directly measure resonance signal changes caused by bulk refractive index alterations resulting from protein or EV binding^90^. Alternatively, these surfaces can be employed to *amplify* signals such as fluorescence excitation, emission, or scattering on the single particle level (Fig. 7). Plasmon-enhanced fluorescence has been demonstrated to significantly amplify analytical signals^91^, with enhancement factors exceeding 100-fold. This technique is particularly valuable for detecting low-abundance markers and facilitating multiplexed analysis of EVs. For example, utilizing plasmonic gold “nanowell” surfaces has enabled the analysis of small EVs that were previously undetectable by conventional fluorescence imaging^92^.

All-dielectric metasurfaces supporting bound states in the continuum (BICs) enhance electromagnetic fields without using metallic components^93^. These approaches have been effectively used for both surface-enhanced fluorescence and surface-enhanced Raman spectroscopy, offering an alternative to traditional metal-based plasmonic structures. In general, photonic crystal resonances have demonstrated promise for enhancing fluorescence signals^88^, with measured enhancements of up to 20. Silicon-nitride metasurfaces, optically transparent, can overcome some limitations of metal-based systems, such as fluorescent quenching and high optical losses. Other metasurfaces, consisting of silicon nanostructures integrated into optical fiber tips^87^, have been designed to improve the sensitivity of fluorescence-based biosensors. These metasurfaces enhanced both the excitation of fluorophores and the collection of emitted photons by the optical fiber, achieving an enhancement factor of up to three orders of magnitudes. Dielectric nanophotonic platforms such as dielectric nanohole arrays have been designed and implemented to detect bulk signals from EVs^25,26^. However, further investigations are needed to assess whether the high Q-factors provided by dielectric platforms over plasmonic resonances^94^ can provide an advantage in fluorescence enhancement for single EV analysis.

The advantage of resonantly enhanced, plasmonic or dielectric, single particle imaging is that the higher signal-to-noise ratios provided by these approaches compared to non-enhanced microscopy enable accurate imaging of EV markers using standard fluorescence microscopes using even relatively low magnification objectives. Therefore, a statistically relevant number of hundreds or even thousands of particles can be imaged at once, while the most sensitive non-enhanced imaging approaches, i.e., super-resolution techniques, require scanning/stitching and, therefore, more time and complex procedures to analyze a similar number of particles.

Multiplexed marker analysis remains a challenge in single-particle techniques due to the inherently broad excitation and emission spectra of the required fluorophores. The role of nanophotonic enhancement in reducing spectral spillover via selective enhancement is still an understudied area with great potential to increase the number of markers that can be detected in single EV analyses. Plasmonic nanohole arrays exhibit a broad spectral resonance range, enabling the simultaneous enhancement of multiple fluorescence channels. However, the efficacy of enhancement within blue wavelengths is constrained by the inherent optical losses associated with metallic surfaces in that spectral region. Conversely, dielectric metasurfaces offer a promising alternative approach for multichannel fluorescence enhancement: (i) substrate materials, such as silicon nitride, are transparent even in the blue wavelength ranges, and (ii) dielectric arrays can be designed to support multiple high-Q resonances at different wavelengths, simultaneously enhancing multi-color signals.

## Conclusions and outlook

Imaging-based nanophotonic sensing and interferometric label-free imaging offer promise for advancing bioanalytics and clinical diagnostics. Exploiting the unique properties of dielectric and plasmonic metasurfaces, these platforms provide high sensitivity, resolution, and throughput in both bulk and single-particle analyses: (i) plasmonic platforms excel in near-field enhancement, making them well-suited for small molecule detection, and ii) dielectric systems exhibit superior Q-factors and reduced photothermal effects, which is advantageous for detecting larger analytes (e.g., EVs). A significant boost in sensitivity is anticipated through innovative metasurface design, such as optimizing the overlap between near-field regions and analytes within high-Q resonant structures. Besides optical engineering, addressing the following aspects will promote the practical implementation and broader adoption of these technologies.

### Scaling up device fabrication

Metasurface chips are usually fabricated through electron-beam lithography for rapid prototyping. However, this method is unsuitable for manufacturing due to its low throughput and high cost. Transitioning to parallel patterning techniques, such as nanoimprint lithography^95^ or interference lithography^96^, will enable the fabrication of nanoscale features across large substrate areas, facilitating cost-effective and scalable device production.

### Surface treatment

Metasurface chips require surface modification to become compatible with biological fluids^3^. These modifications typically serve two key functions: (i) minimizing non-specific biomolecule adsorption (e.g., protein layers) to mitigate alterations in the metasurface’s optical properties and (ii) introducing chemical groups for conjugating affinity ligands (e.g., antibodies). Grafting polyethylene glycol (PEG) is a promising approach^22^. PEG coatings resist protein adsorption through strong water retention^97,98^, and the molecules can present heterobifunctional moieties^69^, one for surface attachment and the other for bioconjugation. Implementing PEG-coating in metasurface devices will be facilitated by establishing PEGylation protocols tailored to diverse substrates (e.g., Au, SiO_2_, Si_3_N_4_). Controlling PEG composition, such as the ratio of long and short PEG chains, will also be essential to ensure efficient target capture while minimizing non-specific interactions^99^. The surface-independent, bio-inspired polydopamine coating approach is a less specific yet more versatile and efficient solution, recently applied to sensing with dielectric surfaces^71^.

### Integration with machine learning

Machine learning (ML) can streamline the processing of large datasets generated by nanophotonic and label-free microscopy. In EV imaging, for example, ML models can be trained to identify and characterize individual vesicles, facilitating the discovery of EV subpopulations and enabling population-scale statistical analysis; similar ML approaches have proven valuable in advancing cellular analysis^100–103^. A crucial initial step is creating well-annotated datasets to train robust ML models.

### Evaluation with clinical samples

The inherent complexity of clinical samples poses analytical challenges for metasurface-based biosensors. For instance, human plasma contains over 1000 distinct protein species whose concentrations span twelve orders of magnitude in dynamic range^104,105^. Abundant matrix components can compromise sensor performance through various mechanisms, including increased non-specific binding, alterations to the local refractive index, and a congested imaging field. Sensor evaluation thus requires using matrix-matched samples, such as plasma spiked with target analytes, to replicate intended analytical conditions. This approach will enable a rigorous assessment of sensor reliability and robustness and, importantly, facilitate the optimization of sample preprocessing protocols for enhanced analytical performance.

These advancements will make metasurface platforms a powerful analytical tool in research and clinical settings. The platforms’ exquisite sensitivity will enable the detection of low-abundance analytes, aiding early disease diagnosis and personalized medicine. Furthermore, the high-throughput capabilities of these platforms will streamline large-scale studies, accelerating scientific discovery. Ultimately, integrating these technologies into routine clinical practice will contribute to improved patient outcomes and a deeper understanding of biological processes.
